# Adherence of doctors to hypertension clinical guidelines in academy charity teaching hospital, Khartoum, Sudan

**DOI:** 10.1186/s12913-019-4140-z

**Published:** 2019-05-14

**Authors:** Hiba Salah Abdelgadir, Maisa Mustafa Elfadul, Nisreen Haider Hamid, Mounkaila Noma

**Affiliations:** 1 Family Medicine, Public and Tropical Health Program, University of Medical Sciences and Technology, P.O. Box: 12810, Khartoum, Sudan; 2 Public and Tropical Health Program, University of Medical Sciences and Technology, P.O. Box: 12810, Khartoum, Sudan; 3 Research Methodology & Biostatistics, University of Medical Sciences and Technology, P.O. Box: 12810, Khartoum, Sudan

**Keywords:** Doctors, Adherence, Hypertension, Clinical guidelines, Clinical governance, Sudan

## Abstract

**Background:**

Clinical guidelines are systematically proven statements that help physicians to make healthcare decisions for specific medical conditions. Non-adherence to clinical guidelines is believed to contribute significantly to poor delivery of clinical care, and hence poor clinical outcomes. This study aimed at investigating adherence of doctors to hypertension clinical guidelines in Academy Charity Teaching Hospital, Khartoum, Sudan.

**Methods:**

A cross-sectional hospital-based study was conducted during the period from January 2017 to October 2017 on a sample of 150 doctors. Adherence of doctors to hypertension guidelines was measured through the modified JNC7 adherence tool. Descriptive statistics was used to summarize the data (mean, standard deviation, median) and analyzed by frequency tables. Chi square test used to determine association among categorized variables. Logistic regression analysis was conducted to determine the relation between adherence to hypertension guidelines and the explanatory variables. All statistical tests were considered statistically significant when *p value* < 0.05.

**Results:**

Of the 150 participants, 92% (138/150) were aware of the major hypertension treatment guidelines. 71% (98/138) reported the use of guidelines recommendations in their practice. Whereas 52% (78/150) were aware of local Sudanese guidelines. High adherence rate was highly statistically associated with job titles (*p* = 0.000), and also associated with age of the participants (*p* = 0.024) and duration of clinical experience (*p* = 0.012). However, the logistic regression analysis revealed despite all those variables were contributing to adherence to the treatment guidelines, only duration of clinical experience was statistically significant (*p* = 0.022).

**Conclusion:**

The overall adherence of doctors to hypertension treatment guidelines was very low. This study highlights how a gap in clinical governance contributes to low adherence to clinical guidelines. Establishing regular clinical audit, issuing regulations to enforce the use of updated guidelines, along with introducing training programs in hospitals and continuous assessment to the practicing doctors are suggested as crucial interventions. Considerable efforts to build clinical governance in Sudan are required.

## Background

High blood pressure (BP) is a widespread disease [1]. Globally, it is estimated that the number of hypertensive patients exceeds one billion, and it is expected to reach 1.56 billion by 2025, resulting in about a 60% increase compared to the year 2000 [2, 3]. About 9.4 million deaths occur annually as a result of hypertension (HTN) and its complications [4]. In the recent years, HTN has also been considered as a cause of about 50% of mortality in cases of stroke and heart disease [5, 6]. Worldwide, numerous guidelines have been issued for hypertension management to be used as reference criteria for the practicing doctors [7].

The institutions which have developed guidelines include Joint National Committee (JNC) on Detection, Evaluation, and Treatment of High Blood Pressure [3, 8, 9], National Institute for Health and Care Excellence (NICE) [10], European Society of Hypertension (ESH)/European Society of Cardiology (ESC) [11] and American Society of Hypertension/International Society of hypertension (ASH/ISH) [12]. The most recent published guidelines for hypertension management is the JNC 8, which is considered standard guidelines in many clinical situations [9]. In Sudan, the second edition of the local Sudanese hypertension guidelines was issued in 2014 by the Sudanese Society of Hypertension (SSH) [13]. Despite the development of these guidelines, many doctors still treat hypertensive patients according to their preference and clinical experience [14, 15].

Different blood pressure goals and pharmacological treatment options are recommended by the available guidelines according to the clinical need of each patient [16]. Globally, the first line antihypertensive drugs vary from diuretics, angiotensin-converting enzyme inhibitors (ACEI), angiotensin II receptor blockers (ARBs) and calcium channel blockers (CCB) [17–19]. The first line antihypertensive drugs as recommended by the JNC 8 guidelines in 2014 include both CCB and ACEI, as well as diuretics [9].

In developing countries, absence of clinical governance and regular clinical audit is thought to be the main reason of non-adherence to clinical guidelines. Clinical governance delivers safety context and quality assurance [20]. Extensive efforts are required to build, clearly define and raise doctor’s awareness about clinical governance in developing countries [21–23]. Policy makers must integrate point of care testing with clinical management protocols to ensure that clinical governance is well implemented. Adherence to guidelines and standards, managerial and clinical stakeholders are required to make sure point of care testing is cost effective, safe and clinically justified [20].

Adherence of doctors to clinical guidelines has been studied in various situations [15, 24, 25], however, their adherence to hypertension guidelines remain scarce. In Sudan, this is the first documented study that measures adherence of doctors to hypertension guidelines in line with the local and international guidelines. It aims at assessing the awareness of doctors about the hypertension guidelines, measuring their adherence rates and determining the barriers to adherence. This study is of great importance to provide baseline data on non-adherence to the guidelines, the causes and possible solutions to assist related decision and policy makers in developing appropriate evidence-based strategies and implement appropriate health programs for the benefit of both the patients and the care givers.

## Methods

### Study design and population

A descriptive cross-sectional hospital-based study was conducted in the Academy Charity Teaching Hospital (ACTH), Khartoum/Sudan, during the period from January 2017 to October 2017 on a purposive convenient sample of 160 medical doctors. Ten doctors refused to participate in the study. Those who participated were Physicians/Consultants (*n* = 13), Registrars (*n* = 72), Medical Officers (*n* = 24) and House Officers (*n* = 41) working at ACTH and in charge of management of adult hypertensive patients. They were distributed in the departments of Internal medicine, Family medicine, Emergency medicine and the Intensive Care Unit. Doctors, practicing in other departments other than the ones above-mentioned were excluded. Data were collected through a standardized pre-coded structured questionnaire. Before starting the survey, a pilot study was conducted on a randomly selected ten doctors from the targeted study population. This helped revising the wording of some questions to be more understandable for the respondents. Collected data included demographic characteristics, awareness and adherence to hypertension guidelines, causes of non-adherence and rates of adherence to guidelines based on a modified scoring system [26].

### Modified scoring tool and weighting criteria

A modified Joint National Committee (JNC 7) adherence tool [26] was used to adapt the research instrument to Sudan context. The modifications were the exclusion of the criteria on drug therapy, observations of the practices of doctors, reviewing of patient records, and thus developing a flexible scoring system that allows the examination of adherence to any of the hypertension guidelines using doctors self-reporting system. The modified JNC 7 instrument included thirteen criteria which were code either 1 or 2 based on the importance of a given criterion on the outcome being measured. Six criteria were considered weighting high and were each coded 2. They were (i) appropriate pharmacological treatment for patients with other diseases, (ii) for uncontrolled HTN increase the dose or change the drug, medical laboratory tests including yearly measurements of: (iii) low-density lipoprotein (LDL), high-density lipoprotein (HDL), triglycerides (TG) and cholesterol, (iv) serum creatinine, (v) blood glucose and (vi) performing electrocardiogram (ECG). The seven remaining criteria were each coded 1 and they were (i) assessing major cardiovascular risk factors, (ii) use a recommended blood pressure goal, (iii) follow up of uncontrolled hypertension within 4 weeks of the last visit, (iv) always discussing life style recommendations, medical laboratory tests including yearly measurements of: (v) hematocrit, (vi) potassium, and (vii) calcium levels. In the overall, a total score 19 was obtained based on the thirteen criteria. Two adherence groups were then created, low adherence for participants whose total score ranged from 0 to 11, and high adherence for those scoring between 12 to 19. According to the weighting system described above, low adherence had a value of < 60% and high adherence a value of ≥60%. Cronbach Alpha test was done to measure the reliability of the respondent’s answers to the self-administrated questionnaire. The answers of the respondents tested through Cronbach’s alpha were under the true of value of reliability (ranging from 0.70 to 0.95) of a survey for thirteen items of the scoring system with a value of alpha of 0.762, that reflects high consistency and reliability of the questionnaire.

### Statistical analysis

The statistical package for social sciences (SPSS 23) was used to summarize the data numerically (mean, standard deviation, median) and graphically (frequency tables). Associations between categorical variables were determined through the Chi-squared (χ^2^) tests. A binary logistic regression analysis was performed to determine the relationship between adherence to hypertension guidelines and its associated factors. All statistical tests were considered as significant when *p-value* < 0.05.

## Results

Of the 150 participants, 58.0% (87/150) were females, 84.7% (127/150) of the participants were aged between 21 to 41 years and 66.7% (100/150) have been practicing for 1 to 5 years. Table 1 revealed their respective position and on job training.

**Table 1:** Demographic characteristics of the participants, (*n* = 150)

Variables	Number	%
Age groups:		
21–30 years	63	42.0
31–41 years	64	42.7
≥ 42 years	23	15.3
Gender:		
Female	87	58.0
Male	63	42.0
Duration of clinical experience:		
1–5 years	100	66.7
6–10 years	32	21.3
≥ 11 years	18	12.0
Job titles:		
House officer	41	27.3
Medical officer	24	16.0
Registrar	72	48.0
Consultant/Physician	13	8.7
Professional Training:		
Internal Medicine	99	66.0
Family Medicine	30	20.0
Emergency Medicine	16	10.7
Intensive Care Unit	5	3.3

The majority 92.0% (138/150) of the participants were aware of the major hypertension treatment guidelines and 71.0% (98/138) were practicing based on the hypertension treatment guidelines. Of those practicing based on the treatment guidelines, 58.1% (57/98) reported regularly following updated editions of the guidelines. Less than half 48.0% (72/150) were not aware of the Sudanese hypertension guidelines. Reasons reported for not using Sudanese guidelines are lack of access *70.7% (55/69), low utilization 13.0% (9/69) and irregular* updates of the local guidelines 7.3% (5/69). Concerning adherence to guidelines, of the 150 participants, only 38.0% (57/150) highly adhered to the guidelines, the remaining 62.0% (93/150) scored low adherence (Table 2).

**Table 2:** Frequency distribution of the participants according to their awareness, and adherence to the hypertension guidelines

Characteristics	Number	%
Awareness of major hypertension treatment guidelines (*n* = 150):		
Aware	138	92.0
Not aware	12	8.0
Following certain hypertension treatment guidelines (*n* = 138):		
Following	98	71.0
Not following	40	29.0
Following of updated guidelines editions (*n* = 98)		
Following	57	58.1
Not following	42	42.9
Awareness of local Sudanese hypertension guidelines (*n* = 150):		
Aware	78	52.0
Not aware	72	48.0
Reasons of not using the local Sudanese guidelines (*n* = 69):		
Lack of accessibility	55	79.7
Low utilization	9	13.0
Irregular updates of the local guidelines	5	7.3
Adherence of doctors to the hypertension guidelines (scoring system results) (*n* = 150):		
High adherence	57	38.0
Low adherence	93	62.0
Suggestions to increase guidelines adherence rates (*n* = 150):		
Regular training programs	85	56.7
Continuous assessment	38	25.3
Imposing a local hospital treatment protocol	27	18.0

The two predominately followed guidelines were the JNC guidelines and the NICE guidelines 46.9% (46/98) and 34.7% (34/98) respectively, followed in rank by the Sudanese HTN guidelines 9.2% (9/98) and the American HTN guidelines 8.2% (8/98). The European HTN guidelines was reported to be used by one participant 1.0% (1/98), as displayed by Table 3.

**Table 3:** Frequency distribution of participants according to the followed guidelines, (*n* = 98)

Guidelines	Number	%
JNC guidelines	46	46.9
NICE guidelines	34	34.7
Sudanese HTN guidelines	9	9.2
American HTN guidelines	8	8.2
European HTN guidelines	1	1.0

A multiple-choice question on adherence to guidelines revealed the barriers which hampered participants from adhering to them. The most reported barrier was the inaccessibility (not available) as reported by 65.3% (98/150) of the participants, followed by pressure from patients 45.3% (68/150) and the amount of information contained in guidelines 32.7% (49/150). Lack of resources was reported by 30.7% (46/150), and other limiting factors were lack of agreement with specific guidelines 8.7% (13/150), lack of outcome expectancy 8.7% (13/150), lack of self-efficacy 8.7% (13/150) lack of agreement with guidelines in general 3.3% (5/150) and lack of time 7.3% (11/150), as revealed by Table 4.

**Table 4:** Barriers to adherence to the guidelines, (*n* = 150)

Characteristics	Number	%
Guideline inaccessibility	98	65.3
Pressure from patients	68	45.3
Large volume of the guideline information	49	32.7
Lack of resources	46	30.7
Lack of agreement with specific guidelines	13	8.7
Lack of agreement with guidelines in general	5	3.3
Lack of outcome expectancy	13	8.7
Lack of time	11	7.3
Lack of self-efficacy	13	8.7

A statistically significant association was found between adherence to the hypertension guidelines with age of doctors (*p value* = 0.024), duration of clinical experience (*p value* = 0.012), and job titles (*p value* = 0.000) as displayed by Table 5.

**Table 5:** Association between demographic characteristics of the participants and adherence to the hypertension guidelines, (*n* = 150)

	Adherence		
Variables	Low adherent	Highly adherent	*P*-value
Age groups:			
21–30 years	47	16	0.024
31–41 years	33	31	
≥ 42 years	13	10	
Duration of clinical experience:			
1–5 years	69	31	
6–10 years	18	14	0.012
≥ 11 years	6	12	
Job titles:			
House officer	35	6	
Medical officer	17	7	
Registrar	36	36	0.000
Consultant/Physician	5	8	

A binary logistic regression analysis was carried out to assess the relationship between adherence to hypertension guidelines and age of the participants, their years of work experience and the job title. The reliability of the model was 64.0%. The model predicted perfectly adherence. Work experience in years which was contributing to the model by 0.119 was statistically significant with a *p-value* of 0.022. Age of the participants and job title were all not statically significant with a *p-value* of respectively 0.65 and 0.164, they were contributing to the model by receptively 0.131 and 0.389, age was contributing with by 1.14 (95 CI: 0.647–2.01) and job title by 1.5 times (95% CI: 0.853–2.55), as displayed by Table 6.

**Table 6:** Logistic regression model predicting adherence based on age, years of working experience and job title of the study participants (*n* = 150)

Variables in the Equation	B	Wald	df	*p-value*	Odds Ratio	95% C.I. for OR	
						Lower	Upper
Age in years	0.131	0.207	1	0.65	1.14	0.647	2.01
Work experience in years	0.119	5.228	1	0.022	1.127	1.017	1.248
Job title	0.389	1.937	1	0.164	1.475	0.853	2.55
Constant	−2.324	13.39	1	0	0.098		

## Discussion

Our findings indicated that the participants had a high level (92.0%) of awareness of the hypertension guidelines. The use of these guidelines in clinical practice was reported to be 71.0%, however, 58.1% of the participants used updated guidelines. These results were in line with data published by Barbouni et al. [27]. Caution was pointed out by a study in Cyprus which found a significant difference between doctor practices and the European guidelines [28].

Results of our study pointed out the tendency of doctors to comply with international guidelines rather than the local Sudanese guidelines which also exist. Reasons to explain this tendency might be (i) the non-introduction of local guidelines in post graduate studies and training programs in Sudan; (ii) the lack of familiarity with the local guidelines and (iii) their inaccessibility because of restricted dissemination. This appeals for raising awareness about the local guidelines to enhance their use, and pursuing their regular update.

Although many doctors reported that they were using hypertension guidelines in their daily practice, their adherence to hypertension clinical guidelines was found to be very low (38%), which implies that their clinical practices were not in line with the new guideline recommendations. Adherence of doctors to hypertension guidelines varied with the geographical location as reported elsewhere in the literature. Adedeji et al. reported in their study an adherence rate of 51.9% [15], while an adherence rate of 53.5% to the JNC7 was published by Ardery el al. [29].

Lack of accessibility was the most frequent barrier to adherence (65.3%) in our study. This was confirmed by Echlin et al. who also reported, lack of accessibility, time, guideline instruction and critical appraisal ability as barriers to adherence to clinical guidelines [30]. On the other hand, Lugtenberg et al. reported lack of agreement with the recommendations as the most observed barrier, followed by environmental factors and lack of knowledge regarding the guidelines recommendations [31].

A limitation of our research was related to its restriction to one of the seven teaching hospitals of Khartoum State. Another limit was the purposive convenient sampling used to select the study participants. Nonetheless, as prospective cross-sectional study, our findings revealed that adherence to hypertension guidelines can be predicted based on the explanatory variables which were namely age of the doctors, their work experience years and job position held. Our findings were strengthened by McKinlay et al. study which concluded that length of clinical experience and type of patients affect adherence to the guidelines [32]. In addition, low adherence rates were associated with old age of doctors and less clinical experience as published elsewhere [9]. Finally, our study pointed out that imposing local hospital treatment protocols, regular training programs and regular doctor assessments might increase adherence rates. Such a conclusion was also made by Lugtenberg et al. who emphasized that continuous medical education might increase adherence of doctors to guidelines [31].

## Conclusion

This study highlights a gap in clinical governance which contributes to low adherence to clinical guidelines. Our study appeals for issuance of regulations and protocols to ensure the use of updated guidelines, along with the introduction of training programs in hospitals and the commencement of continuous assessment that would aid in increasing adherence rates and eventually improve patient outcomes. Establishing regular clinical audits to strengthen the governance system is crucial for all of the concerned entities.

## Abbreviations

ACEI: Angiotensin-converting enzyme inhibitors
ACTH: Academy Charity Teaching Hospital
ARBs: Angiotensin II Receptor Blockers
BP: Blood pressure
CCB: Calcium channel blockers
ECG: Electrocardiogram
HDL: High-density lipoprotein
HTN: Hypertension
JNC: Joint National Committee
LDL: Low-density lipoprotein
NICE: National Institute for Care and Health Excellence
SSH: Sudanese Society of Hypertension
TG: Triglycerides
UMST: University Medical Sciences and Technology
